# Hippo signaling in mammalian reproduction

**DOI:** 10.1530/REP-25-0016

**Published:** 2025-05-30

**Authors:** Robin E Kruger, Farina Aziz, Amy Ralston

**Affiliations:** ^1^ Cell and Molecular Biology Graduate Program, Michigan State University, East Lansing, Michigan, USA; ^2^ Reproductive and Developmental Sciences Training Program, Michigan State University, East Lansing, Michigan, USA; ^3^ Department of Biochemistry and Molecular Biology, Michigan State University, East Lansing, Michigan, USA

**Keywords:** preimplantation, ovary, spermatogenesis, decidualization, trophoblast reproductive disease, Hippo

## Abstract

The Hippo signaling pathway, so named for its massive overgrowth mutant phenotypes, has become one of the most exciting signaling pathways to emerge in the field of reproductive biology. While disruption of Hippo is associated with tumorigenesis in many organs and tissues, relatively less is understood about the normal roles of Hippo signaling in the reproductive organs. Here, we highlight the recent literature illuminating the roles of Hippo pathway members in mouse and human reproduction. We place special emphasis on the inputs and outputs of Hippo signaling during preimplantation development, where Hippo signaling has been extensively studied in both mouse and human. We note a common emerging theme is the critical and highly conserved role of Hippo signaling in epithelia of the reproductive organs. We also discuss human reproductive disorders, whose etiology may be related to dysregulation of Hippo signaling, and possible therapies that have been proposed to correct this dysregulation. Finally, we describe the edge of our knowledge, which currently limits our understanding of Hippo signaling in reproductive health and disease.

## The Hippo pathway defined

The Hippo signaling pathway is a highly conserved pathway that is essential for animal development and adult homeostasis. The pathway is named for overgrowth, reminiscent of a hippopotamus, observed in fruit flies carrying Hippo pathway mutations (Harvey *et al.* 2003). Subsequent studies using biochemical and genetic approaches have uncovered core components and mechanisms of Hippo signaling, including upstream inputs and downstream outputs. Interestingly, a large variety of signals can act upstream to initiate Hippo signaling, including extracellular ligands, steroids, stress, and mechanical cues (Zhong *et al.* 2024).

In most contexts, the major output of Hippo signaling is modulation of gene expression. The mechanisms by which Hippo transcriptional targets are specifically regulated are an active area of investigation. In general, Hippo signaling represses the transcriptional activity of the paralogous transcriptional co-factors YAP1 and WWTR1 (also called TAZ) by preventing their association with TEAD family DNA-binding factors. In the absence of Hippo signaling, YAP1 and WWTR1 associate with TEADs, and the YAP1/WWTR1/TEAD complex then induces transcriptional changes. The YAP1/WWTR1/TEAD complex is generally thought to act as a transcriptional activator. However, YAP1 and WWTR1 can also repress transcription by recruiting transcriptional repressors, for example, of the RUNX, PPAR, SMAD, or VGLL families, or the Nucleosome Remodeling and Deacetylation (NuRD) complex (Ferrigno *et al.* 2002, Hong *et al.* 2005, Beyer *et al.* 2013, Varelas 2014, Kim *et al.* 2015, Valencia-Sama *et al.* 2015, Cotton *et al.* 2017, Zhang *et al.* 2018). The clinical implications of this are that, depending on molecular context, the YAP1/WWTR1/TEAD complex could possess both tumor suppressor and oncogenic activities (Kim *et al.* 2018).

Several excellent review articles have described roles for Hippo in homeostasis, disease, development, and regeneration in model organisms and in humans (Moya & Halder 2016, Misra & Irvine 2018, Davis & Tapon 2019, Zheng & Pan 2019). Yet, the roles of Hippo signaling in reproduction have been less comprehensively summarized. Drawing from studies in mice and humans, we review known roles for Hippo signaling in mammalian reproduction, beginning with the early embryo and then proceeding upstream to explore paternal and maternal Hippo roles in germ cell production, pregnancy, and disease.

## Hippo signaling and the early embryo

For more than 15 years, the mouse embryo has served as a paradigm for uncovering the roles of Hippo signaling in mammalian reproductive biology. In this context, Hippo signaling governs the first cell fate decision that occurs during mouse embryogenesis: separation of fetal and placental fates. This cell fate decision occurs among the initially identical blastomeres during morula stages. This Hippo-regulated cell fate decision is a crucial juncture in the establishment of pregnancy.

**Figure 1:**
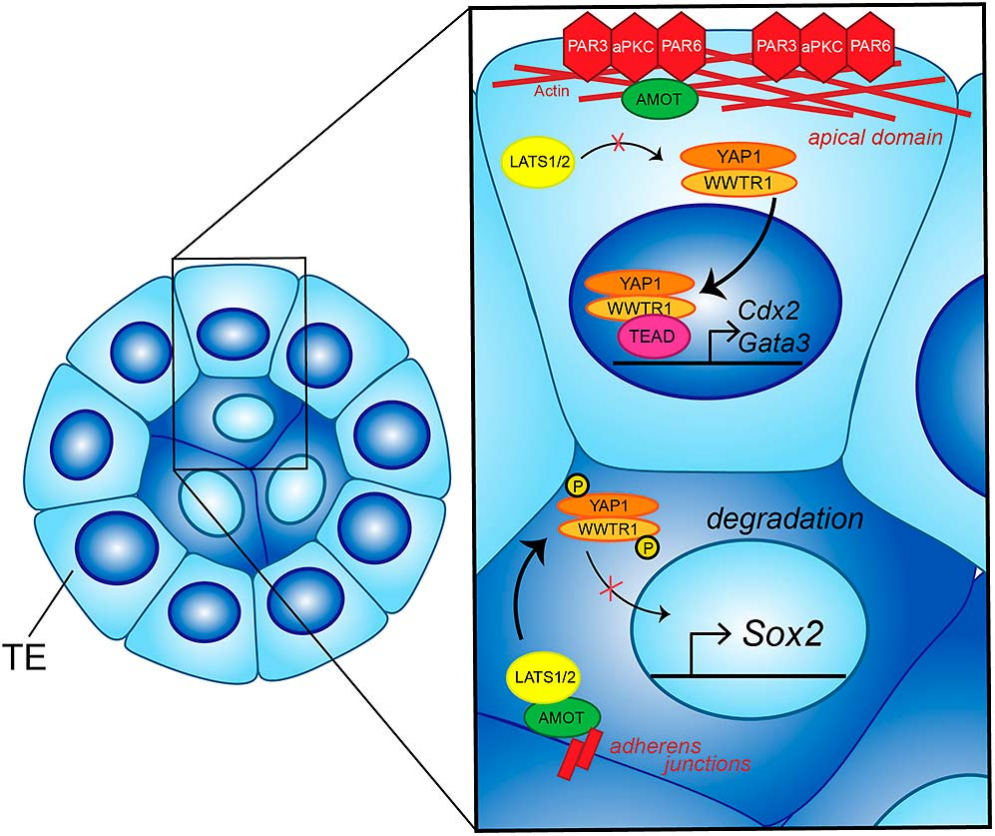
Hippo signaling regulates cell fate specification in the mammalian early embryo. Starting around the 16-cell stage, angiomotin (AMOT) is localized to the symmetrically distributed adherens junctions, enabling activation of Hippo kinases LATS1/2. By contrast, trophectoderm (TE) cells are polarized along the radial axis, leading to AMOT tethering in the apical domain and rendering LATS1/2 inactive. In inside cells, active LATS1/2 phosphorylate transcriptional co-factors YAP1/WWTR1, which leads to their cytoplasmic retention and degradation. Meanwhile, in TE cells, YAP1/WWTR1 are free to enter the nucleus, where either partners with DNA binding protein TEAD4 to activate expression of *Cdx2* and *Gata3*, and repress expression of *Sox2*.

Around the 16-cell stage, Hippo signaling becomes active only in the inner cells, which are fated to contribute to the fetus (Nishioka *et al.* 2009). In this context, active Hippo signaling means that LATS1/2 kinases actively phosphorylate YAP1/WWTR1, which leads to their cytoplasmic retention and/or degradation. Thus, the YAP1/WWTR1/TEAD4 transcriptional complex does not assemble in inner cells (Fig. 1). By contrast, Hippo signaling is silenced in the outer trophectoderm (TE) cells of the morula, which are fated to become placenta. This is achieved by virtue of TE cell polarization because key Hippo signaling components become sequestered within the apical domain, rendering LATS1/2 kinases inactive in TE cells (Hirate *et al.* 2013, Leung & Zernicka-Goetz 2013). As a consequence of inactive Hippo signaling, the YAP1/WWTR1/TEAD4 transcriptional complex forms in the nucleus and then drives expression of TE-specific genes *Cdx2* and *Gata3*, while repressing expression of the fetal lineage marker *Sox2* (Yagi *et al.* 2007, Nishioka *et al.* 2009, 2008, Ralston *et al.* 2010, Wicklow *et al.* 2014, Frum *et al.* 2019, 2018). In fact, embryonic cells lacking *Yap1* and *Wwtr1* can express markers of both TE and fetal lineage, leading to cell death (Frum *et al.* 2018). Thus, in the early embryo, Hippo signaling is responsible for resolving cell fate conflicts, acting downstream of epithelialization.

Most intriguingly, the embryonic roles and regulation of Hippo signaling have been shown to be highly conserved in mammals. In cow, pig, and human embryos, as in mice, Hippo signaling is repressed in the trophectoderm, presumably allowing YAP1/WWTR1/TEAD4 to promote expression of the TE gene *GATA3* and to repress expression of *SOX2* (Emura *et al.* 2020, Gerri *et al.* 2023). In mice, *Yap1* and *Wwtr1* are both maternal-effect genes, meaning that the oocyte contributes YAP1 and WWTR1 proteins, which function during embryogenesis (Frum *et al.* 2019, 2018). In fact, YAP1 has been implicated as a key factor in activating the zygotic genome in early murine embryos (Yu *et al.* 2016). These observations strongly suggest that Hippo signaling plays earlier roles in reproduction, starting in the ovary.

## Hippo signaling in the ovary

Processes critical to female reproduction, including oogenesis, ovulation, and production of female sex hormones, occur in the ovaries. The ovary is organized into follicles, the essential unit for ovulation. Each follicle has a single germ cell at its center, which matures during ovulation, and several surrounding layers of somatic cells, including theca cells and granulosa cells. Epithelial granulosa cells provide the oocyte with nutrients and growth factors and, once mature, are responsible for estrogen production. Mesenchymal theca cells produce androgens for granulosa cells to convert into estrogens. Following ovulation, both somatic cell types luteinize to form the corpus luteum, a temporary endocrine structure that produces progesterone during early pregnancy. Altogether, the follicle is highly dynamic, with intricate interactions and coordinated maturation among cell types, suggesting tightly regulated intercellular signaling. Failure of any of these cell types to communicate properly will result in oocyte death, failed ovulation, or pregnancy loss.

The Hippo pathway has been shown to play an essential role in female fertility, particularly through regulation of ovarian cell growth, follicle maturation, and key ovarian functions, such as ovulation and steroidogenesis. Changes in Hippo signaling have been associated with defects in ovarian function, such as polycystic ovarian syndrome (Li *et al.* 2012, Kawamura *et al.* 2013, Jiang *et al.* 2017, Maas *et al.* 2018), primary ovarian insufficiency (Ye *et al.* 2017), and simple age-related ovarian functional decline (Li *et al.* 2015). Understanding the mechanisms by which Hippo signaling normally regulates ovarian function is therefore a high priority for female reproductive health.

**Figure 2:**
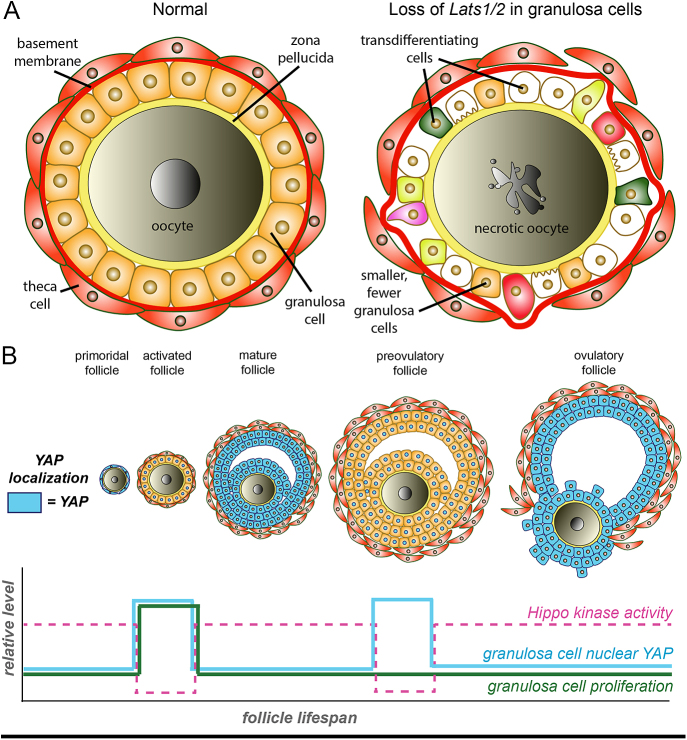
Ovarian function requires dynamic Hippo signaling in somatic cells. (A) When Hippo signaling is experimentally repressed in granulosa cells, follicles suffer adverse effects such as loss and transdifferentiation of granulosa cells and oocyte death. (B) Requirements for Hippo signaling in granulosa cells change through the life of a follicle. In primordial follicles, Hippo signaling activity is high, restricting YAP1 to the cytoplasm and preventing premature follicle growth. At activation, Hippo signaling in granulosa cells decreases to allow YAP1 to enter the nucleus and induce follicle growth. As the follicle enters maturity before ovulation, nuclear YAP1 decreases again in granulosa cells, before spiking in preovulatory follicles and then dropping dramatically in response to the LH surge at ovulation. ‘Hippo kinase’ refers to LATS1/2 and MST1/2.

Evidence from mice suggests that a balance of Hippo signaling activity is needed for proper ovarian function. Both YAP1 inactivation and hyperactivation result in ovarian dysfunction *in vivo* (St John *et al.* 1999, Sun *et al.* 2015, Ji *et al.* 2017, Ye *et al.* 2017, Lv *et al.* 2019, 2020, Hu *et al.* 2019, Sun & Diaz 2019, Tsoi *et al.* 2019). Interestingly, granulosa cells appear to be particularly sensitive to Hippo activity levels, since both LATS activity and the activity of YAP1/WWTR1 in the ovary are more essential in granulosa cells than in the oocyte itself (Abbassi *et al.* 2016, Yu *et al.* 2016). For example, too little Hippo activity in granulosa cells, as in the loss of *Lats1/2*, causes granulosa cells to transdifferentiate into a variety of cell types, especially osteoblasts (Fig. 2A, Tsoi *et al.* 2019), leading to tumor formation (St John *et al.* 1999, Sun *et al.* 2015).

It is not yet clear why granulosa cells are so sensitive to HIPPO levels. One possibility is that the requirements for Hippo activity change over the lifespan of a follicle (Fig. 2B), a model that is supported by multiple lines of evidence. Early follicles possess elevated Hippo kinase (LATS1/2 and MST1/2) activity, which, in turn, decreases YAP1 entry into granulosa cell nuclei. Thus, Hippo activity helps prevent premature follicle activation (Sun *et al.* 2015, Xiang *et al.* 2015, Hu *et al.* 2019, Lv *et al.* 2019, De Roo *et al.* 2020), restricts granulosa cell proliferation, and maintains granulosa cell identity (Sun *et al.* 2015, Tsoi *et al.* 2019). As follicles begin to mature, Hippo signaling then becomes transiently inactivated to allow YAP1 to induce granulosa cell proliferation and maturation (Li *et al.* 2015). Consistent with this, loss of *Yap1* in mature granulosa cells produced no functional or morphological defects, whereas loss of *Yap1* in proliferating granulosa cells caused decreased ovary size and follicle number (Lv *et al.* 2019). At the preovulatory stage, Hippo activity (again MST1/2 and LATS1/2) becomes attenuated, allowing nuclear YAP1 to upregulate expression of genes that respond to luteinizing hormone (LH), preparing the follicle for ovulation (Ji *et al.* 2017, Sun & Diaz 2019, Godin *et al.* 2022). After ovulation, and possibly in response to the LH surge, Hippo signaling then reactivates, limiting proliferation and inducing corpus luteum formation. Therefore, Hippo activity is dynamic, and its levels must be carefully regulated in granulosa cells throughout the follicle lifespan.

Fascinating unanswered questions remain. It is still unknown what acts upstream of Hippo signaling activity in granulosa cells and how this mechanism is modulated throughout follicle maturation. One possibility that has been proposed is mechanical signals, since force is a known input into Hippo signaling (Hsueh *et al.* 2015). In the primordial follicle, the high tissue stiffness of the outer ovarian cortex could induce Hippo activity, leading to YAP1 phosphorylation and nuclear exclusion. As the maturing follicles move inward toward the less stiff ovarian medulla, Hippo activity would decrease, allowing YAP1 nuclear entry. In support of this hypothesis, isolated strips of ovarian tissue display lower levels of phosphorylated YAP1 (pYAP1), and folliculogenesis appears more advanced near the edges of the strip, where the ECM has been disrupted (Grosbois & Demeestere 2018).

Finally, our knowledge of Hippo signaling in the oocyte is also incomplete. Oocyte YAP1 and WWTR1 are both required for early embryogenesis (Frum *et al.* 2018). Yet, YAP1 protein localization is strictly cytoplasmic in the oocyte, and *Yap1* is dispensable in the oocyte for follicle formation, follicular maturation, and ovulation (Sun *et al.* 2015, Abbassi *et al.* 2016, Yu *et al.* 2016, Hu *et al.* 2019). By contrast, WWTR1 appears to be strongly nuclear in the oocyte (Sun *et al.* 2015). However, no oocyte-specific knockouts of *Wwtr1* in the ovary have been examined to date, nor have oocyte-specific knockouts of *Lats* or other Hippo pathway members. Additional study is required to pinpoint exactly when and where Hippo signaling is active in the normal and diseased female germline.

## Hippo signaling in diseases of the ovary

Hippo signaling dysregulation has been implicated in several ovarian pathologies, particularly polycystic ovarian syndrome (PCOS). However, at this stage, most evidence for a role of Hippo signaling components in PCOS is correlative. For example, sequence polymorphisms in the *YAP1* coding region have been correlated with PCOS by genome-wide association studies (GWAS) (Li *et al.* 2012), implicating, but not proving, a role for *YAP1* in the disease. Similarly, granulosa cells from PCOS patients exhibit variation in the expression of Hippo pathway genes (Li *et al.* 2012, Ji *et al.* 2017, Maas *et al.* 2018). Because Hippo signaling regulates aromatase expression in granulosa cells (Fu *et al.* 2014), it is tempting to speculate that aberrant Hippo signaling could interfere with the conversion of androgens to estradiol, providing a possible mechanism for hyperandrogenism observed in PCOS patients. However, no direct evidence exists tying Hippo dysregulation to manifestation of PCOS in humans (Maas *et al.* 2018, Xia & Du 2022).

Despite incomplete understanding of the etiology of PCOS, the therapeutic modulation of ovarian Hippo signaling can restore fertility in PCOS patients (Kawamura *et al.* 2013). For example, disruptive surgical treatments for PCOS, such as wedge resection and laser drilling, which stimulate F-actin formation and disrupt Hippo signaling, increase follicle growth and have even led to successful births (Kawamura *et al.* 2013, Cheng *et al.* 2015). However, inactivation of Hippo by disruption may also cause quicker depletion of the patient’s ovarian reserve by activating too many follicles too quickly. More recent studies have therefore developed methods to activate follicles more slowly. A combination of simple mechanical compression and incubation with mTOR inhibitors can decrease the number of activated follicles in patient ovarian tissue (Grosbois & Demeestere 2018, Hsueh & Kawamura 2020). The role of Hippo signaling in PCOS, primary ovarian insufficiency, and ovarian cancers, including consideration of possible upstream and downstream mechanisms, is described further in several excellent review articles (Clark *et al.* 2022, Zhu *et al.* 2023).

## Hippo signaling in the uterus

In addition to its roles in ovarian disorders, emerging evidence implicates Hippo signaling dysfunction in uterine disorders, including endometriosis, endometrial fibrosis, and uterine cancers (Liu *et al.* 2013, Song *et al.* 2016, Zhan *et al.* 2016, Pei *et al.* 2019, Zhu *et al.* 2019). To understand the role of Hippo signaling in uterine disease etiology, it is important to first understand the normal functions of Hippo signaling in the uterus. To date, the role of Hippo signaling has been most studied during uterine decidualization.

During early pregnancy, the uterine endometrium undergoes dramatic morphological and functional remodeling to accommodate the implanting blastocyst, a process known as decidualization. Decidualization is critical for the success of a pregnancy, and impairment to this process leads to a multitude of pregnancy disorders, including infertility and miscarriage (Okada *et al.* 2018). One of the hallmarks of decidualization is the differentiation of elongated, fibroblast-like endometrial stromal cells to rounded, epithelial-like decidual cells. These cells secrete hormones, such as prolactin, which are fundamental for embryo implantation via endometrial invasion of trophectoderm-derived trophoblasts.

The role of the Hippo signaling pathway during decidualization has been investigated, initially in cell lines and more recently *in vivo*. Both *Yap1* and *Wwtr1* are required for decidualization in cultured cells (Strakova *et al.* 2010, Chen *et al.* 2017) and in the mouse uterus (Moldovan *et al.* 2024), although the mechanistic connection between Hippo and decidualization is still enigmatic. Interestingly, transcriptional analysis of uteri lacking *Yap1* and *Wwtr1* uncovered pathway annotations connected to endometriosis (Moldovan *et al.* 2024). These observations are consistent with a growing body of evidence connecting Hippo signaling pathway dysregulation to endometrial disease.

## Hippo disruption may lead to development of endometrial disorders

In addition to normal roles in decidualization, YAP1/WWTR1 dysregulation has been associated with endometriosis and endometrial fibrosis (Song *et al.* 2016, Pei *et al.* 2019, 2022, Zhu *et al.* 2019). Both YAP1 mRNA and protein are elevated in endometrial stem cells derived from women with endometriosis (Song *et al.* 2016), consistent with pathological function. In addition, the YAP1/TEAD1 complex increased proliferation and decreased autophagy of endometrial stromal cells derived from women with endometriosis (Pei *et al.* 2019, 2022). These observations suggest that disrupted Hippo signaling/YAP1 overactivation cause overproliferation of endometrial cells in endometriosis. Moreover, the phosphorylation, and therefore cytoplasmic retention, of WWTR1 leads to downregulation of fibrotic gene expression in endometrial fibrosis, consistent with YAP1/WWTR1 hyperactivity in the etiology of this disease (Zhu *et al.* 2019). These observations implicate disruption of Hippo activity as a driver of endometrial disorders, but more research is needed to thoroughly define this relationship. Elucidating the precise functions of Hippo signaling in the uterus during gestation is also important since uterine tissues contribute to placentation, described next.

## Hippo signaling and placentation

The Hippo pathway has been implicated in complications that arise relatively late in pregnancy, including preeclampsia, intrauterine growth restriction, recurrent pregnancy loss, and preterm birth (Sun *et al.* 2018, Saha *et al.* 2020, Hu *et al.* 2022, Ray *et al.* 2022). These observations suggest critical roles for Hippo signaling in placentation. The placenta is a critical exchange interface between mother and fetus and is thus comprised of cells derived from both individuals. During preeclampsia, trophoblast invasion into maternal endometrium is frequently superficial (Fisher 2015), bringing the spotlight onto the role of Hippo signaling in trophoblast invasion.

**Figure 3:**
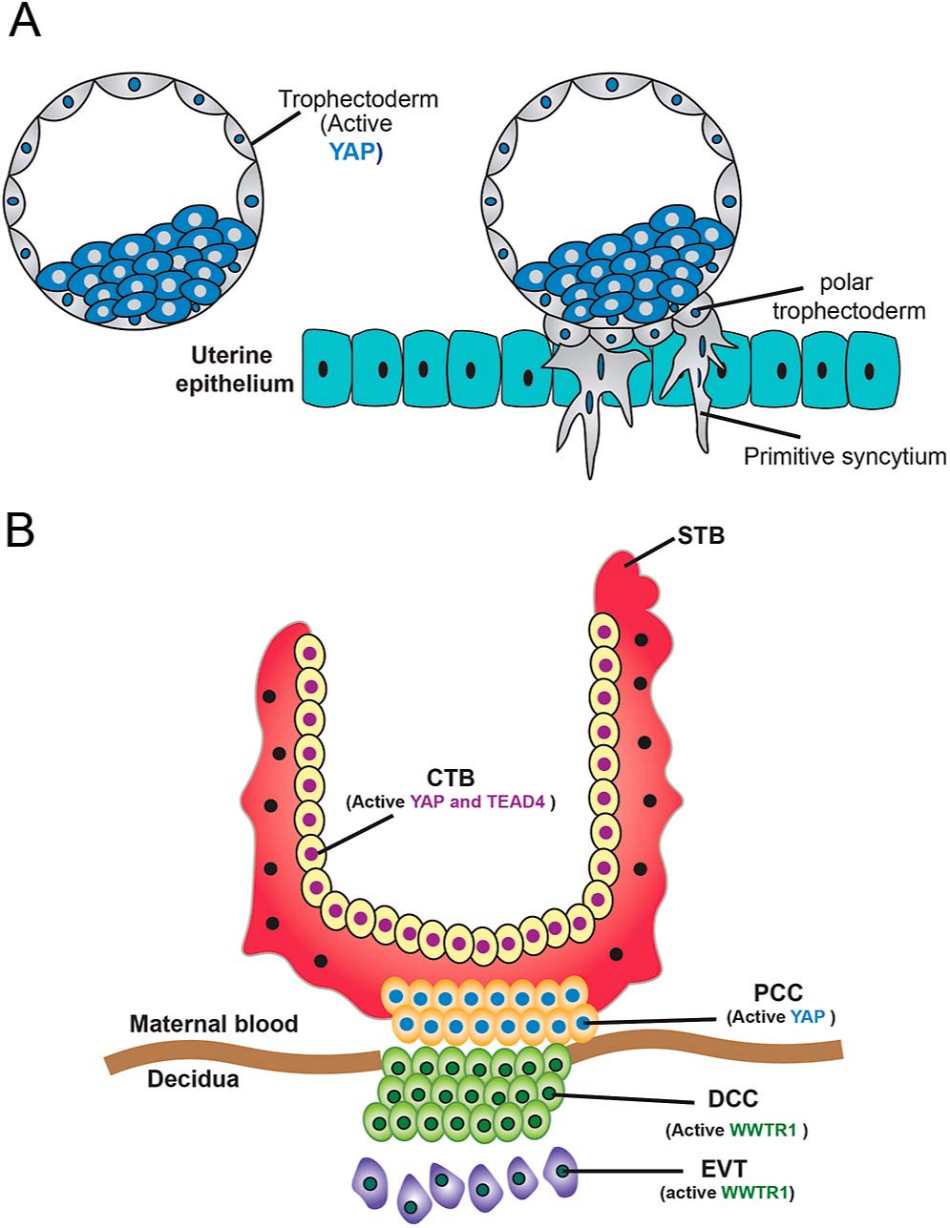
The dynamic changes of Hippo signaling components during TE expansion and establishment of chorionic villi. (A) In the trophectoderm (TE), YAP1 is nuclear, whereas it is cytoplasmic in the inner cell mass (ICM). During implantation, the polar TE makes contact with and invades the uterine epithelium. (B) The unique spatial distribution of Hippo pathway components in different trophoblast subtypes balances self-renewal and differentiation during trophoblast development. CTB, cytotrophoblast; STB, syncytiotrophoblasts; pCC, proximal cell column; dCC, distal cell column; EVT, extravillous trophoblast.

Trophoblast invasion of the uterine endometrium is initiated during implantation (Fig. 3A) and involves precisely coordinated trophoblast cell differentiation and proliferation (Hemberger *et al.* 2020). In this regard, YAP1/WWTR1/TEAD4 have been shown to help maintain the stem cell-like properties of mouse and human trophoblast cells (Nishioka *et al.* 2009, Meinhardt *et al.* 2020, Saha *et al.* 2020, Ray *et al.* 2022), enabling their continued proliferation and multilineage potential (Mizutani *et al.* 2022).

In addition, Hippo pathway members are also thought to promote trophoblast invasion in humans. In support of this, in the first trimester human placenta, TEAD4 and YAP1 show strong overlapping expression in the nuclei of proliferative villous cytotrophoblasts, whereas WWTR1 is abundant in the nuclei of extravillous trophoblasts (Fig. 3B) (Meinhardt *et al.* 2020, Saha *et al.* 2020, Ray *et al.* 2022). Moreover, overexpression of YAP1 in the human trophoblast cell line HTR-8/SVneo led to increased invasive ability of the cells, whereas knockdown of *Yap1* yielded the opposite outcome (Sun *et al.* 2018). Interestingly, the human Vestigial-like transcription factor VGLL1, proposed to play a role in placenta (Sonnemann *et al.* 2023), has recently been shown to partner with TEAD4 in human trophoblast stem cell models (Yang *et al.* 2024). However, whether VGLL1 competes with YAP1/WWTR1 or whether these proteins are regulated by Hippo kinases is still unknown. Additional discussion of the roles of Hippo in pregnancy complications can be found in a recent comprehensive review (Lin *et al.* 2023).

## Hippo signaling in male reproduction

Our review would not be complete without discussion of Hippo roles in spermatogenesis. In fact, the Hippo study has been extensively studied in this context. Studies in mice have demonstrated that Hippo signaling is critically involved in the regulation of spermatogenesis (St John *et al.* 1999, Hossain *et al.* 2007). Spermatogenesis takes place within the seminiferous tubules of the testes, where immature, self-renewing spermatogonial stem cells (SSCs) reside along the inner edge of the tubule. SSCs gradually mature as they move inward toward the tubule lumen. SSCs first differentiate into spermatogonia, which can divide by mitosis to produce meiotically capable spermatocytes. After completing meiosis, haploid spermatocytes differentiate further into spermatids and then undergo morphological changes to become mature sperm in the process of spermiogenesis. Finally, the mature spermatid enters the lumen of the seminiferous tubule as a fully developed spermatozoon. Spermatozoa travel from the seminiferous tubule to the epididymis, where they undergo final maturation before ejaculation.

Several models, including knockouts of *Lats1, Lats2*, *Yap1,* and *Wwtr1,* report decreased testis size (St John *et al.* 1999, Levasseur *et al.* 2017, Abou Nader *et al.* 2022). However, although YAP1 and WWTR1 protein are expressed and localized to the nucleus in male germ cells after puberty (Levasseur *et al.* 2017), the direct, cell-autonomous effect of Hippo signaling on spermatogenesis appears to be limited. Conditional knockout of *Yap1* in mouse spermatogonial germ cells had no apparent effect on the expression of germ cell markers, SSC formation, or sperm count *in vivo* (Abou Nader *et al.* 2019).

**Figure 4:**
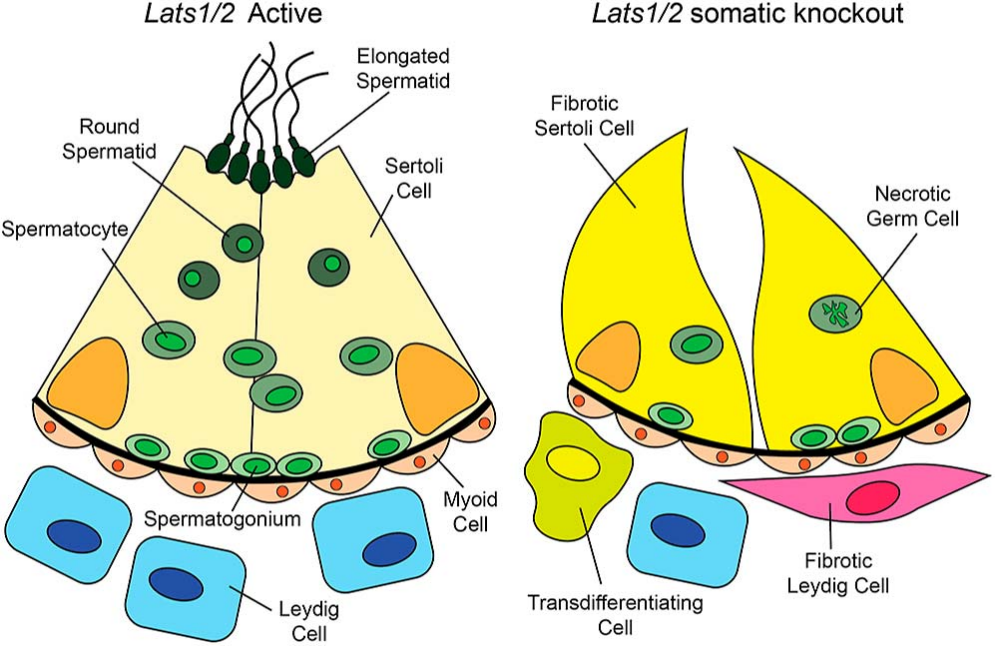
Testicular function requires Hippo signaling in somatic cells. When Hippo signaling is experimentally repressed in Sertoli cells, seminiferous tubules suffer adverse effects such as tissue fibrosis and germ cell death.

Although Hippo signaling is apparently dispensable in germ cells, it is essential to maintain the cell identities of somatic cells of the testis (Fig. 4). This is reminiscent of the role in ovary somatic cells described above. The somatic cells of the testis, Sertoli cells, Leydig cells, and structural interstitial cells, are essential to the process of spermatogenesis. Leydig cells function primarily to produce testosterone, which, among several other targets, is received by Sertoli cells. Sertoli cells act as ‘nurse’ cells for the developing germ cells within the seminiferous tubules, providing hormones, nutrients, structural support, and aiding in waste removal. Proper function of Sertoli and Leydig cells is essential for spermatogenesis and male fertility.

Interestingly, Hippo signaling appears to be most important in Sertoli and Leydig cells for spermatogenesis. Specific knockout of *Lats1* and *Lats2* in Sertoli and Leydig cells led to smaller and disorganized testes as early as embryonic day E14.5 (Abou Nader *et al.* 2022). These knockouts showed decreased expression of many Sertoli cell markers, including *Sox9*. On the other hand, conditional double knockout of *Yap1* and *Wwtr1* in Sertoli cells decreased expression of male-specific genes such as *Dhh, Dmrt1, Sox9*, and *Wt1* at pre-pubertal stages (Levasseur *et al.* 2017). Notably, the loss of cell identity in Sertoli and Leydig cells did not result in sex reversal, as neither *Yap1/Wwtr1* knockout nor *Lats1/2* knockout resulted in upregulation of granulosa cell genes *in vivo* (Levasseur *et al.* 2017, Abou Nader *et al.* 2022).

Ultimately, the requirement for Hippo signaling in somatic cell identity leads to an essential, non-cell-autonomous role in spermatogenesis (Sen Sharma & Majumdar 2017, Abou Nader *et al.* 2022). Conditional double knockout of *Lats1* and *Lats2* in mouse Sertoli cells results in small, disorganized testes with very few observable seminiferous tubules (Abou Nader *et al.* 2022). In addition, germ cells in this model were mostly apoptotic by E17.5, suggesting that active Hippo signaling in Sertoli cells is specifically required for early spermatogenesis. This conclusion is consistent with the finding that knockout of *Yap1* and *Wwtr1* in Sertoli cells does not impair early spermatogenesis, since *Lats1/2* normally prevents the transcriptional activity of YAP1 and WWTR1 (Levasseur *et al.* 2017).

Altogether, these studies show that Hippo signaling is required in male reproductive somatic cells for proper regulation of spermatogenesis and male fertility. However, the specific transcriptional targets of YAP1/WWTR1 and exact regulatory mechanisms remain to be identified. Several lines of evidence suggest that Hippo signaling is required for stabilization of cilia (Hossain *et al.* 2007, Shi *et al.* 2023). This possibility is intriguing, as patients with autosomal dominant polycystic kidney disease, which affects cilia formation, also commonly display male infertility and sperm motility defects (Shi *et al.* 2023).

## Outlook

Surveying the roles of Hippo signaling broadly across reproductive systems, an unexpected theme has emerged. That is, the dysregulation of Hippo signaling tends to result in cell fate defects in diverse contexts, including the preimplantation embryo, and both male and female germ lines. Whether this is coincidental or due to a shared downstream mechanism awaits further study. Identification of YAP1/WWTR1/TEAD transcriptional targets in each setting could help illuminate this question. Moreover, discovering mechanisms downstream of YAP1/WWTR1/TEAD will also aid in the development of therapeutic strategies for treating reproductive diseases that are caused by Hippo signaling dysregulation.

## Declaration of interest

The authors declare that there is no conflict of interest that could be perceived as prejudicing the impartiality of the research reported.

## Funding

R K has been supported by NIHhttps://doi.org/10.13039/100000002 grants T32 HD087166 and T32 DK071212. F A is supported by NIH grant R01 HD108722 to A R.

## Author contribution statement

A R conceived the paper. R K, F A, and A R wrote the paper.
